# monarchr: an R package for querying biomedical knowledge graphs

**DOI:** 10.1093/bioinformatics/btaf549

**Published:** 2025-09-26

**Authors:** Shawn T O’Neil, Brian M Schilder, Kevin Schaper, Corey Cox, Daniel Korn, Sarah Gehrke, Christopher J Mungall, Melissa A Haendel

**Affiliations:** Department of Genetics, University of North Carolina at Chapel Hill, Chapel Hill, NC 27599, United States; Simons Center for Quantitative Biology, Cold Spring Harbor Laboratory, Cold Spring Harbor, NY 11724, United States; Department of Genetics, University of North Carolina at Chapel Hill, Chapel Hill, NC 27599, United States; Department of Genetics, University of North Carolina at Chapel Hill, Chapel Hill, NC 27599, United States; Department of Genetics, University of North Carolina at Chapel Hill, Chapel Hill, NC 27599, United States; Department of Genetics, University of North Carolina at Chapel Hill, Chapel Hill, NC 27599, United States; Division of Environmental Genomics and Systems Biology, Lawrence Berkeley National Laboratory, Berkeley, CA 94720, United States; Department of Genetics, University of North Carolina at Chapel Hill, Chapel Hill, NC 27599, United States

## Abstract

**Summary:**

Biomedical knowledge graphs (KGs) aggregate and provide a wealth of information, linking genes and their variants, diseases, phenotypes, and much more. While these data are available in raw and API-hosted form, to date, functionality for working with KGs in the R programming language has been limited. We introduce monarchr, a package for querying and manipulating KG data. Support for the expansive Monarch Initiative KG is built in, and monarchr can accommodate any KG in the Knowledge Graph eXchange (KGX) format. This tidy-inspired interface offers researchers an intuitive, iterative approach to querying and visualizing KG data.

**Availability and implementation:**

Source code, documentation, and installation instructions are available at https://github.com/monarch-initiative/monarchr.

## 1 Introduction

Knowledge graphs (KGs) are collections of heterogeneous data representing known relationships between entities. They are frequently represented as labeled-property graphs, where both nodes (entities) and edges (relationships) may be annotated with additional attributes (Di Pierro *et al.* 2023). In biomedical contexts, KGs may represent relationships between genes and their molecular functions, diseases and their phenotypes, genetic variants and the diseases they cause, or a combination of these and others. The Monarch Initiative hosts a large biomedical KG for public consumption, the Monarch KG, which includes over 1M entities and 8M relationships across dozens of entity and relationship types. This database is widely used for research, and Monarch provides access via website, the monarch-py Python package, REST API, and other modalities (Putman *et al.* 2024). R is widely used in biomedical applications, including the integration, analysis, and visualization of diverse biological and clinical datasets (Giorgi *et al.* 2022). Here we describe monarchr, an R package for querying both the Monarch and other biomedical KGs. Backed by the tidygraph and igraph libraries (Csardi and Nepusz 2006, Pedersen 2020), monarchr provides a flexible and user-friendly interface for extracting and manipulating KG data.

Graph data structures in general are well supported in R (Butts 2005, Csardi and Nepusz 2006, Pedersen 2020), but packages specific to KGs are few. Related packages include kgraph for constructing KGs from tabular data (Charlton and Yuan 2025), sparql for querying RDF-formatted KGs (Van Hage *et al.* 2013), and Neo2R for querying the popular Neo4j labeled-property graph database (Godard 2024). Some packages are designed for specific KGs: gkgraphR queries the Google KG (Correia 2021), fella utilizes a KG representation of the KEGG database for pathway enrichment (Picart-Armada *et al.* 2018), and DeepTimeKB specializes in KGs for geological sciences (Ma *et al.* 2022). Closely related to (and often incorporated in) KGs are ontologies, which are carefully constructed to allow logical reasoning over hierarchical subclass and other relationships (Smith 2003). R packages focusing on ontologies include ontologyX for importing, manipulating, and plotting ontologies (Greene *et al.* 2017), simona (and ontologyX) for semantic similarity (Gu 2024), ontoFAST for rapid annotation of ontologies (Tarasov *et al.* 2022), and rols for querying the EBI ontology lookup API (Gatto 2013). Beyond generic graph libraries such as NetworkX, Python-based packages for KG access and management are many, often focused on specific applications like embedding or semantic similarity computations (Broscheit *et al.* 2020, Cappelletti *et al.* 2022, Zhapa-Camacho *et al.* 2023). Monarch’s own monarch-py library offers access to node and relationship data via SQLite or Solr backend, returning sets of Entity and Association objects from query lists.

Despite the availability of these tools, there remain several key challenges that monarchr aims to address. Access to the cloud-hosted Monarch Initiative KG is integrated for ease of use, but unlike other KG-specific tools (gkgraphR, fella, and DeepTimeKB), monarchr supports any KG formatted in the Knowledge Graph eXchange (KGX) standard defined by KGHub (Caufield *et al.* 2023). This makes monarchr useful for a wide variety of applications, especially as KGX repositories grow in popularity. While RDF and Neo4j databases provide natural KG storage, these require specialized query syntax (SPARQL and Cypher, respectively) that can be challenging and unintuitive for programmers with limited exposure to these languages. Finally, ontology-only tools such as simona, ontoFAST, and ontologyX are not applicable to more general KGs. While packages like tidygraph, igraph, and kgraph support general graph operations, many KG-specific operations such as property-based neighborhood searches and transitive queries require cumbersome implementations. In contrast, monarchr enables sophisticated queries, interactive exploration, and dynamic visualization for both Neo4j-hosted and file-based KGs via a composable and tidy-inspired interface (Wickham 2023).

## 2 Implementation

### 2.1 Engines

KGs supported by monarchr must conform to the KGX format, which requires nodes to have single-valued id (e.g. “MONDO:0019391”) and multi-valued category (e.g. c(“biolink:Entity”, “biolink:Gene”)) attributes (Caufield *et al.* 2023). While it is a relatively new standard, KGHub’s registry catalogs over 40 biomedical KGs in KGX format as of 2025 and provides a venue for KG sharing. Edges are directed and required to have single-valued subject, predicate, and object attributes (e.g. “MONDO:0019391”, “biolink:has_phenotype”, and “HP:0004322”). As allowed by KGX, nodes and edges may have additional (single- or multi-value) attributes such as name or description, listed by summary() described below.

Access to KG data is mediated by an engine abstraction, with support for two kinds of engines: a file_engine ingesting KGX-TSV .tar.gz files, and a neo4j_engine for connecting to Neo4j databases. Engines provide additional features beyond mediating KG access. Both engine types support preferences, allowing the definition of a primary category, or pcategory, for nodes. The KGX standard supports multiple category entries as a multi-valued list, but does not specify an ordering or “primary” category. For example, Noonan syndrome (MONDO:0018997) has categories biolink:BiologicalEntity, biolink:Disease, biolink:NamedThing, and others. The default set of preferences, designed for Biolink-compatible KGs, specifies biolink:Disease as one of a set of preferred categories defining the node’s pcategory. This dramatically simplifies operations over groups of nodes (e.g. extracting/counting the number of disease nodes and gene nodes) without the need for extensive user-defined wrapper functions. These are adjustable and described in the *Engine Preferences* vignette.

Neo4j engines provide features specific to remote data access patterns, including query pagination and optional result caching for the duration of the R session, boosting speed and performance. Finally, a Monarch-specific monarch_engine is provided, subclassing the neo4j_engine with additional features provided by the Monarch Initiative API such as free-text search (Putman *et al.* 2024). Engines are simply established as file_engine(filename), neo4j_engine(url), and monarch_engine(), with optional parameters for preferences and other features. This makes it exceedingly simple to reuse scripts with different KGs by swapping out the engine.

### 2.2 Queries

All engines provide two crucial functions: fetch_nodes() and expand(). The former is used to fetch an initial set of nodes (but no edges) as a local (in-memory) graph. The latter takes such a graph and expands it to include additional neighboring nodes and edges from the backing KG. All results (local graphs) are returned as tbl_kgx objects, which extend tbl_graph objects from the tidygraph package supporting user-friendly node- and edge-table representations. Although only engines interact with backing KG data, returned tbl_kgx graphs keep track of the engine that produced them, allowing additional expansions using R pipe operators. Consider the following example:

## Initialize engine

monarch_engine() |>

 ## Fetch Noonan and Alstrom syndrome nodes

 fetch_nodes(query_ids = c(“MONDO:0018997”,

       “MONDO:0008763”)) |>

 ## Expand to include phenotypes

 expand(predicates =“biolink:has_phenotype”)

Here, an instantiated engine is first sent to fetch_nodes(), returning a graph with two nodes and no edges. Next, expand() uses the graph’s attached engine to further pull all adjacent edges with predicate biolink:has_phenotype, resulting in a graph with the original nodes connected to newly added edges and phenotype nodes. The expand function is expected to take a local graph, and always returns a supergraph of its input.

The fetch_nodes() function allows fetching by node identifier as illustrated above, or via a logical expression over node attributes to fetch nodes in bulk (including regular expression matching). For example, biolink:Gene nodes in the Monarch KG have an additional in_taxon_label attribute. The following code fetches all human genes:

monarch_engine() |>

## Get all human genes

fetch_nodes(in_taxon_label == “Homo sapiens” & “biolink:Gene” %in_list% category)

Although node category is multi-valued, we do not override %in%, which implements different semantics when applied to multi-valued (list) data in standard R (entry %in% a_list does not indicate which values of a_list contain entry).

While expand() does not support logical expressions over arbitrary edge or edge attributes, several parameters allow defining which nodes and edges are included in the expansion. Users may specify a set of edge predicates to follow, and/or a set of node categories to restrict to, and/or the direction of edges to follow.

As discussed earlier, many KGs subsume ontologies, where transitive relationships play a significant role. Directional, transitive expansion is thus supported by expand() as well; fetching the subtype hierarchy for Noonan syndrome (MONDO:0018997) is as simple as:

monarch_engine() |>

 ## Get the initial disease node

 fetch_nodes(query_ids = “MONDO:0018997”) |>

 ## Get disease subtypes

 expand(predicates = “biolink:subclass_of”,

      direction = “in”,

      transitive = TRUE)

 ## or use descendants()

Convenience functions descendants() and ancestors() are provided for transitive inward and outward biolink:subclass_of expansions, respectively. Repeated (but not fully transitive) expansions are supported with expand_n(); while descendants() includes all subclass nodes, expand_n(predicates = “biolink:subclass_of”, direction = “in”, n = 2) includes only two levels of subclasses. Together, these functions allow users to grow networks easily and precisely.

### 2.3 Exploration, visualization, and other features

KGs are frequently complex: the Monarch KG utilizes over 100 distinct node categories and two dozen relationship predicates. While name and description are common optional attributes, different node and edge types include more specialized attributes, such as in_taxon_label for biolink:Gene nodes and frequency_qualifier for biolink:has_phenotype edges. The Biolink data model is well-documented (Unni *et al.* 2022), but in practice this complexity presents challenges in effective KG exploration and use.

To support exploration, an engine’s summary() lists available node categories, edge predicates, and node and edge properties. It also silently returns a list of this information, as well as a named list of available categories and predicates, which can be used for auto-completion in RStudio. Beyond basic counts, sampling strategies also support exploration, but a random sample is unlikely to illustrate the diversity of information available. Instead, engines provide an example_graph() function, which fetches a sample of nodes and edges guaranteed to represent every available node category and edge predicate. We refer interested readers to the *Exploring Knowledge Graphs* vignette for details and examples.

Graphs in monarchr inherit from tidygraph, which in turn inherit from igraph, and are thus compatible with a variety of R network visualization libraries, including ggraph and igraph for static plots, and visNetwork, networkD3, and threejs for interactivity (Network Visualization using “vis.js” Library [R package visNetwork version 2.1.2] 2022, D3 JavaScript Network Graphs from R [R package networkD3 version 0.4.1] 2025; Pedersen 2024, Lewis 2025). The included plot() provides basic visualization via ggraph, and cytoscape() for exporting to the Cytoscape desktop application via the RCy3 library (Gustavsen *et al.* 2019, Shannon *et al.* 2003). The *Visualizing Knowledge Graphs* vignette provides examples.

Other features supported by monarchr include saving graphs in KGX format and functions to perform transitive and related operations. These include rollups with custom aggregation functions, transferring data between nodes over edges (to support e.g. rolling up causal gene names over disease nodes), transitive closures, and transitive reductions. Example usage of these functions is found in the *Rollups and Transitivity* vignette.

## 3 Example: KG-based entity prioritization

Genetic, phenotypic, and related information are often used in biostatistical methods. Phenome-Wide Association Studies (PheWAS), e.g. examine how specific genetic or other variation is associated with a broad range of phenotypes, enabling the discovery of pleiotropic effects, novel genotype–phenotype relationships, and drug targets (Bastarache *et al.* 2022). While this approach is widely used, identifying variants or other features associated with diseases or drugs of interest can be tedious. Similarly, characterizing an appropriate phenome (set of phenotypes) to test can influence study quality and statistical power, especially for rare diseases (Delavan *et al.* 2018, Wan *et al.* 2025). Here we demonstrate how monarchr can be used to identify variants, genes, and phenotypes in support of PheWAS and similar methods. We consider Noonan syndrome, a multisystem genetic disorder involving diverse gene–variant–phenotype relationships (Roberts *et al.* 2013).

**Figure 1.**
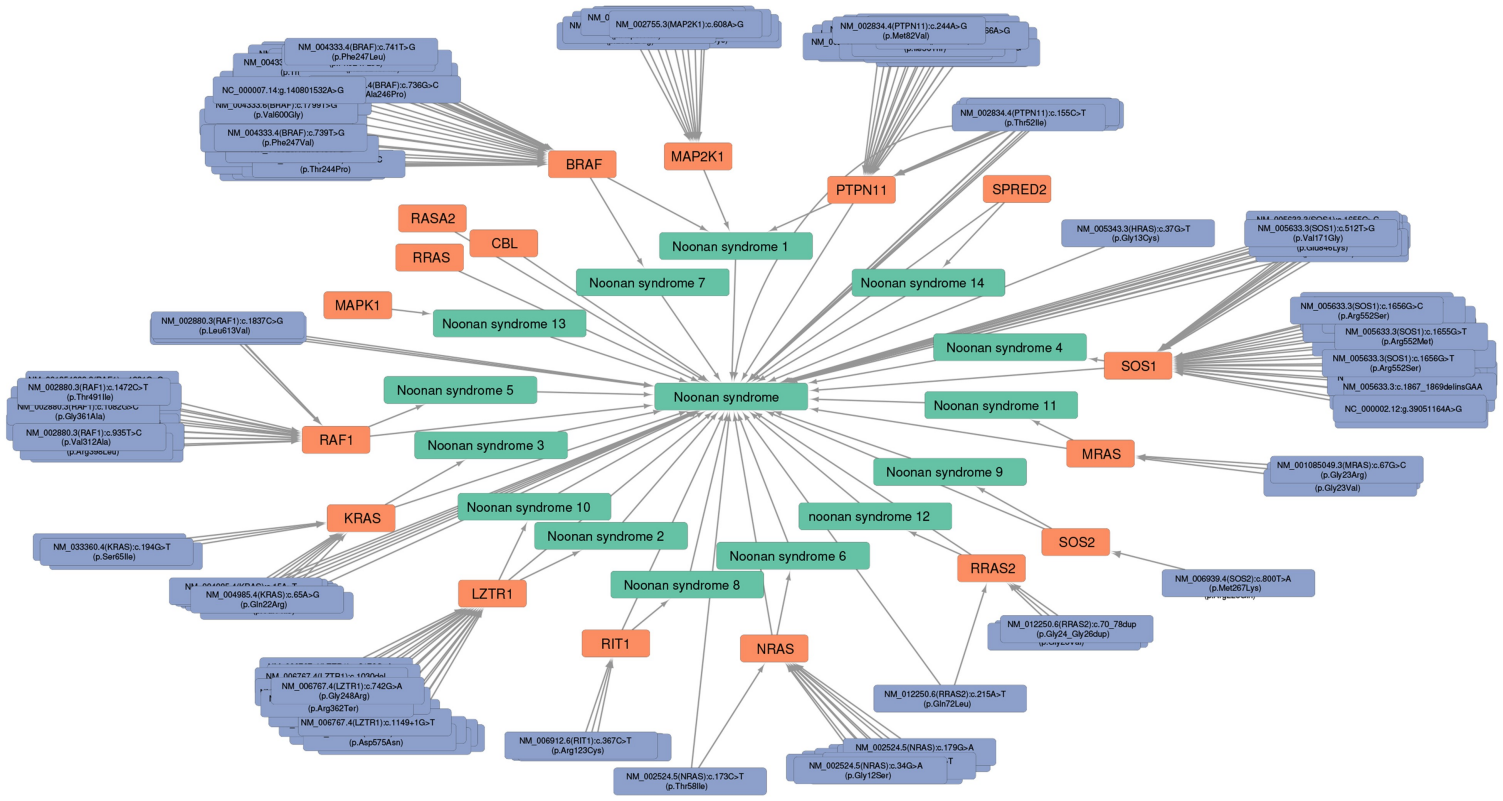
All genetic variants and genes associated with Noonan syndrome or its subtypes. Nodes are colored by pcategory and visualized with cytoscape() followed by adjustments in the Cytoscape GUI. Noonan syndrome and its subtypes are shown in the central ring, genes in middle, and genetic variants at the periphery.

We begin fetching the node for Noonan syndrome (by name here, though note that such a query may match multiple nodes), followed by descendants() equivalent to expand(predicates = “biolink:subclass_of”, transitive = TRUE, direction = “in”). For illustration purposes, we create two expansions: first, sequence variants directly connected to any of these subtypes in any way, and second, sequence variants of genes associated with these subtypes in any way. Note that because Monarch includes data from different sources, some variants are present in both expansions. After producing a union of the two graphs with kg_join() (an implementation of tidygraph’s graph_join() with KG-specific functionality), we visualize it with cytoscape() in Fig. 1.

noonans <- monarch_engine() |>

 fetch_nodes(name ="Noonan syndrome") |>

 descendants()

direct_vars <- noonans |>

 expand(categories =  "biolink:SequenceVariant")

gene_vars <- noonans |>

 expand(categories ="biolink:Gene") |>

 expand(predicates = "biolink:is_sequence_variant_of")

all_vars <- kg_join(direct_vars, gene_vars)

cytoscape(all_vars)

Since “biolink:is_sequence_variant_of” is the only predicate connecting genes and variants in the Monarch KG, all_vars could also be generated as noonans |> expand(categories = “biolink:Gene”) |> expand(categories = “biolink:SequenceVariant”).

Each of these loci represents a potential PheWAS target of study. The Human Phenotype Ontology lists over 18,000 phenotypes (Gargano *et al.* 2024); rather than considering all of them, we can easily fetch only those connected to this set of genes, diseases, and variants. Since the Monarch KG represents phenotypes across many species, we use activate() and filter() from tidygraph and dplyr to keep only those associated with humans (resulting in 771 phenotypes, not shown). Finally, while directly connected phenotypes (e.g. Mitral valve prolapse (HP:0001634)) are likely of highest interest, more generalized phenotypes may be of interest as well (e.g. Abnormal mitral valve morphology (HP:0001633)). Rather than fetch all ancestor phenotypes with ancestors() (the fully transitive complement to descendants()), we fetch an additional two levels above the direct phenotype set with a repeated expansion, before extracting only the nodes in tabular format (2404, also not shown).

direct_phenos <- all_vars |>

 expand(categories = "biolink:PhenotypicFeature") |>

 # tidygraph, activate nodes table

 activate(nodes) |>

 # filter nodes to Human phenotypes

 filter(str_starts(id,"HP:"))

expanded_phenos_table <- direct_phenos |>

 expand_n(predicates ="biolink:subclass_of",

      direction ="out",

      n = 2) |>

 nodes()# extract the nodes dataframe

## 4 Conclusion

Biomedical KGs collate vast amounts of data from diverse sources, but effective use of this information requires tools to match. The monarchr package provides first-class support for the comprehensive Monarch Initiative KG, while also supporting other KGX KGs accessed either as files or hosted in Neo4j labeled-property graphs. Paginated and session-cached queries are fast, fetching up to 1400 nodes per second. Finally, a simple but compositional API supports exploration and analyses, drawing on tidygraph and other R packages for data manipulation and visualization. Planned future work will build on these strengths with support of additional Monarch-specific features such as semantic similarity search and text annotation, and enhanced filtering flexibility in expand().

## Data Availability

Source code for monarchr is available via GitHub at https://github.com/monarch-initiative/monarchr and Zenodo at https://doi.org/10.5281/zenodo.14553217. The Monarch KG is available at https://monarchinitiative.org and https://kghub.org/kg-registry/resource/kg-monarch/kg-monarch.html.
